# Paediatric nuclear medicine practice: an international survey by the IAEA

**DOI:** 10.1007/s00259-019-04624-w

**Published:** 2019-12-08

**Authors:** G. L. Poli, L. Torres, M. Coca, M. Veselinovic, M. Lassmann, H. Delis, F. Fahey

**Affiliations:** 1 Dosimetry and Medical Radiation Physics Section, International Atomic Energy Agency, Vienna, Austria; 2 Department of Nuclear Medicine, DIC, CENTIS, Havana, Cuba; 3 Medscan Nuclear Medicine and PET/CT Center, Concepcion, Chile; 4 Department of Nuclear Medicine, University Hospital Würzburg, Würzburg, Germany; 5 Division of Nuclear Medicine, Boston Children’s Hospital, Boston, Massachusetts USA

**Keywords:** International multi-centre survey, Paediatric nuclear medicine, Administered activity

## Abstract

**Purpose:**

The International Atomic Energy Agency (IAEA) decided to initiate a survey to evaluate the current status of the practice of paediatric nuclear medicine worldwide, with the focus mainly on low and middle-income countries specifically in Latin America, Eastern Europe, Africa and Asia. This investigation sought to determine if the practice in paediatric nuclear medicine in these countries differed from that indicated by the survey of the Nuclear Medicine Global Initiative (NMGI) and if nuclear medicine practitioners were following established paediatric nuclear medicine guidelines.

**Methods:**

A total of 133 institutes took part in the survey from 62 different IAEA member states within Africa (29), Asia (39), Europe (29) and Latin America (36). The four most frequent conventional (single-photon) nuclear medicine procedures were ^99m^Tc labelled MDP, DSMA, MAG3 and pertechnetate thyroid scans. In addition, 46 centres provided data on FDG PET/CT, including exposure data for the CT component. Nearly half of the sites (48%) perform less than 200 paediatric nuclear medicine studies per year, while 11% perform more than 1000 such studies per year.

**Results:**

Administered activities largely exceeded the recommendations for most of the sites for DMSA, MAG3 and pertechnetate, while compliance with international standards was somehow better for MDP studies. For FDG PET, the results were more uniform than for conventional nuclear medicine procedures. However, the use of CT in PET/CT for paediatric nuclear medicine revealed a high variability and, in some cases, high, dose-length product (DLP) values. This observation indicates that further attention is warranted for optimizing clinical practice in FDG PET/CT.

**Conclusions:**

Overall, in most parts of the world, efforts have been undertaken to comply either with the EANM dosage card or with the North American Consensus Guidelines. However, variability in the practice of paediatric nuclear medicine still exists. The results of this survey provide valuable recommendations for a path towards global standardization of determining the amount of activity to be administered to children undergoing nuclear medicine procedures.

## Introduction

Nuclear medicine has been shown to be of considerable value when applied in children including its application in urology, orthopaedics, oncology, endocrinology and neurology. This practice requires the administration of radiopharmaceuticals to children exposing them to low levels of ionizing radiation. In addition, the use of hybrid positron emission tomography (PET) or single-photon emission computed tomography (SPECT) combined with computed tomography (CT) exposes the child to additional radiation. Although there is no direct evidence demonstrating adverse health effects in humans for the levels of radiation exposure associated with medical imaging, many consider it prudent to optimize the exposure to patients receiving these studies. In addition, children are thought to be at a higher risk to ionizing radiation than adults. Therefore, one should pay special attention when these studies involve children.

Although there have been image guidelines and model protocols for adult nuclear medicine examinations for quite some time, until recently, there have not been such guidelines for paediatric nuclear medicine. Within the European Association of Nuclear Medicine (EANM), there has been development on paediatric guidelines in nuclear medicine since the late 1990s leading to the publication of the EANM paediatric dosage card in 2007 [1]. Independently, a North American group led by representatives from the Society of Nuclear Medicine and Molecular Imaging (SNMMI), the Society of Pediatric Radiology (SPR) and the American College of Radiology (ACR) as well as the Image Gently Alliance developed the North American Consensus Guidelines (NACG) for administered activities for children and adolescents in 2011 [2]. In 2014, an effort to harmonize the EANM dosage card and the NACG resulted in the publication of new versions of each [3]. More recently, the North American group published an update to their guidelines in 2016 that included five new procedures [4]. The Japanese Society of Nuclear Medicine has also developed paediatric guidelines [5].

There have been several investigations into the variations in the practice of nuclear medicine in children. In 2007, Treves et al. surveyed 13 dedicated paediatric hospitals in North America as to their approach to tailoring the administered activity in children of different sizes for 16 procedures commonly performed in paediatric nuclear medicine [6]. This study demonstrated considerable variation in the administered activities utilized even within dedicated paediatric institutions. After the publication of the NACG in 2012, a follow-up survey of the same 13 institutions was performed which indicated a reasonable reduction in the variation in the administered activities as well as a general lowering of the amount of administered activities for most of the procedures addressed by the survey [7]. In addition, Fahey et al. performed a survey of general hospitals in USA regarding the practice of paediatric nuclear medicine in 2013 considering the hypothesis that the variations in such practice may be different in general hospitals as compared to dedicated paediatric hospitals which indeed was shown to be the case [8]. In the general hospital survey, responders were asked if they were familiar with the Image Gently campaign, whether they knew about the NACG and whether they had modified their administered activities in children as a result of the guidelines. Of note, 83% of the responding sites were familiar with the Image Gently campaign, 58% were familiar with the NACG and 55% modified their administered activities based on the NACG. Thus, practically all sites that were familiar with the guidelines modified their paediatric nuclear medicine practice as a result. This conclusion was borne out in the reported, estimated activities that the sites would have administered to two hypothetical patients. A survey of administered radioactivity for 13 nuclear medicine procedures commonly performed in paediatric patients was conducted at the university-affiliated hospitals in South Korea in 2013 [9]. This study indicated that all institutions scaled their administered activity for smaller patients, but there was a wide variation in the schema used for such, including the use of the EANM dosage card, the Korean Society of Nuclear Medicine manual and other approaches.

In 2012, 13 international organizations involved in the practice of nuclear medicine formed what was deemed the Nuclear Medicine Global Initiative (NMGI). The underlying objectives of this endeavour were to promote human health by advancing the fields of nuclear medicine and molecular imaging, to encourage global collaboration in education and to harmonize procedure guidelines and other policies that ultimately lead to improvements in quality and safety in the field throughout the world. This group chose its inaugural project “Standardization of Administered Activities in Pediatric Nuclear Medicine”. In this regard, the NMGI published their report in two parts [9, 10]. Part 2 included the results of a global survey demonstrating the variations in the practice of paediatric nuclear medicine worldwide. The results of this survey also indicated the value of having specific, regional paediatric guidelines as those regions that had such (i.e. North America, the EANM and Japan), in general, considerably smaller variations in their practice. As a result, one of the recommendations of the NMGI Project 1 was that countries or regions with no current paediatric nuclear medicine guidelines should either develop their own or officially adopt currently existing ones. However, the authors are not aware of any paediatric nuclear medicine guidelines that have been developed or reported beyond those discussed above.

Although there were over 300 responders to the NMGI survey from 29 countries, very few of these were from the developing world. Therefore, the IAEA within the context of the Coordinated Research Project E2.40.20 on “Evaluation and Optimization of Paediatric Imaging” decided to administer a survey that was very similar to that from the NMGI but to focus this mainly on low- and middle-income countries specifically in Latin America, Eastern Europe, Africa and Asia. This investigation sought to determine if the practice of nuclear medicine in children in these countries differed from that indicated by the NMGI survey and if nuclear medicine practitioners were following any of the established paediatric nuclear medicine guidelines.

## Methods

The format of the survey was based on the one used for the NMGI but modified to suit the needs of this study. The survey involved questions regarding hospital and clinic demographics, their adherence to published or local guidelines, their practice regarding single-photon (planar and SPECT) imaging as well as their PET/CT practice including their use of ^18^F fluorodeoxyglucose (FDG) and the CT component. In addition, the brand and make of the PET/CT scanners were requested. Regarding demographics, the sites were asked the maximum age for which they would consider someone to be a paediatric patient and the approximate number of paediatric nuclear medicine studies performed each year at their institution.

For the single-photon studies, responders were asked to rank the top 5 paediatric nuclear medicine imaging procedures performed at their facility (choosing from a list of 12 procedures, with the option for “other” entries), the guideline that was being followed (if any) for each of these 5 and either the typical patient administered activity or the method of adjusting the administered activity for individual patients. The survey asked for details of administered activity based on the usual operating protocols rather than a collection of details for actual patients. If the site indicated that they scaled the administered activity linearly by weight, they were asked to provide activity per body mass (in either MBq/kg or mCi/kg). Many sites utilize a “minimum” administered activity below which they will consider regardless of how small the patient is. For example, if for a radiopharmaceutical, a patient’s body mass would indicate that she should receive 5 MBq, but the minimum activity for that protocol is set at 15 MBq, then the patient would receive 15 MBq. This limit is meant to assure that there will be enough activity present in the patient for an imaging study of adequate image quality for the clinical task at hand. If a site sets a minimum activity for the procedure in question, they were asked to report this activity in MBq or mCi. Also, if the site had a maximum administered activity for very large patients, it was also reported in MBq or mCi. In addition, the survey inquired about administered activities for a hypothetical 5-year-old boy (20 kg and 110 cm tall) and a 10-year-old girl (30 kg and 140 cm tall). The latter questions regarding the hypothetical patients were used as a quality control check on whether the facility was following an established guideline if they stated they were doing so. For facilities performing PET/CT imaging, the survey asked questions about administered activity for whole body paediatric FDG imaging and details on CT acquisition in the context of PET/CT. With respect to the administered activity of FDG, the sites were asked to provide the same information as with the single-photon radiopharmaceuticals, i.e. were they following an established or local guideline, what was their activity per body mass, the minimum and maximum activity and the activities they would administer to the two hypothetical patients. For the CT component in paediatric PET/CT, sites were asked if they utilized automatic exposure control and whether they acquired a CT for diagnosis or a low-dose CT for attenuation correction. They were also asked to provide the age, weight, tube voltage (in kV_p_) and dose-length product (DLP in mGy⋅cm) for up to their last five paediatric patients imaged with PET/CT in their clinic.

As previously stated, this project was centred mainly on low- and middle-income countries in Latin America, Eastern Europe, Africa and Asia. Contact information came from a variety of sources including data bases of nuclear medicine medical physicists associated with other projects within the IAEA and the NUMDAB [11]. In addition, participants in the CRP project from Latin America collected contact information from additional medical physics colleagues at several regional conferences. The sites considered as potential responders were limited to those countries that are eligible to receive support from the IAEA through the Technical Cooperation Programme. An email was sent to all potential responders either to ensure the accuracy of the contact information or to obtain more up-to-date information. The survey was web based for simplification and ease of data consolidation. Google Forms® (google.com) was used for data collection. The survey was made available to all potential responders in English. The survey was also available in Spanish for institutions from Latin America. For those institutions that agreed to take part to the survey, an email with web links to the survey was sent to the contact person that the site identified as responsible for the survey. The survey was administered between 2018 and 2019 within a span time of 8 months.

Characteristics of the nuclear medicine services as well as use of the imaging procedures are presented using descriptive statistics (percentages, medians and quartile ranges). For the administered activities for the two hypothetical patients, we tabulated the percentage of responses within 20% of the guidelines which were considered to be in basic compliance. The administered activity per body mass, minimum and maximum activities as well as the administered activities for the two hypothetical patients are displayed in box and whisker plots where the median, first and third quartiles and minimum and maximum values are indicated. In cases for which the answers to the survey were incomplete, unclear or presumably inaccurate, the responders were approached a second time for amendments to their initial answer.

## Results

Approximately 700 potential responding institutes were initially contacted, out of which 196 agreed to reply to the survey. The link to the survey was sent to these 196 sites, with a deadline for completion of about 2 months. A total of 133 responses were received (68% response rate) from 62 different member states within Africa (29), Asia and Pacific (39), Europe (29) and Latin America (36) as reported in Table 1.

**Table 1:** List of countries and sites per country

Region	Country	Number of sites
Africa	Algeria	3
	Angola	1
	Cameroon	1
	Egypt	3
	Gabon	1
	Ghana	1
	Kenya	2
	Mauritania	1
	Mauritius	1
	Morocco	3
	Namibia	2
	Niger	1
	Nigeria	1
	South Africa	3
	Tanzania	2
	Tunisia	1
	Zambia	1
	Zimbabwe	1
Asia and Pacific	Bangladesh	1
	Cambodia	1
	India	2
	Indonesia	5
	Iran	1
	Iraq	1
	Malaysia	3
	Myanmar	3
	Nepal	1
	Pakistan	5
	Philippines	3
	Sri Lanka	2
	Thailand	8
	Vietnam	3
Europe	Albania	1
	Armenia	1
	Bosnia and Herzegovina	3
	Bulgaria	1
	Croatia	2
	Cyprus	1
	Estonia	1
	Kazakhstan	1
	Latvia	1
	Lithuania	2
	Macedonia	1
	Malta	1
	Romania	1
	Russia	2
	Serbia	4
	Slovakia	1
	Turkey	5
Latin America	Argentina	6
	Barbados	1
	Bolivia	1
	Brazil	5
	Chile	6
	Colombia	4
	Cuba	2
	El Salvador	1
	Honduras	1
	Mexico	3
	Paraguay	1
	Peru	1
	Uruguay	4
	Total	133

The grouping of countries in different regions is done according to the convention used within the Technical Cooperation Programme of the IAEA.

**Fig. 1:**
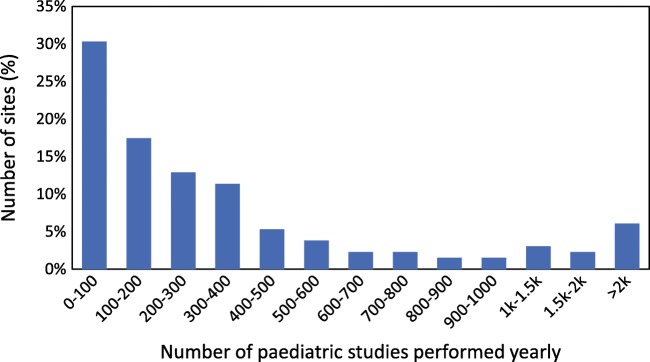
Percentage distribution of the number of paediatric studies performed yearly by the institutes involved in the survey

There was a wide variation in the maximum age considered for paediatric patients ranging from 12 to 20 years of age with a median of 17 years and a most common response of 18 years. In addition, 46 of the sites (35%) indicated that they had PET/CT. Of these with PET/CT, 21 had a scanner from General Electric (46%), 17 from Siemens (37%) and 8 from Philips (17%). A histogram of the number of paediatric studies performed per year at each site is shown in Fig. 1. Nearly half of the sites (48%) perform less than 200 paediatric nuclear medicine studies per year, while 11% perform more than 1000 such studies per year. More than half of the sites (55%) indicated that they have adopted the EANM paediatric dosage card for guidance when performing nuclear medicine procedures in children. In addition, 8% stated that they utilized the NACG, 4% guidelines specified by the local nuclear medicine society, 31% utilized a combination of these, while 2% used guidelines developed independently. For FDG PET, more than half of the 46 sites indicated that they utilized the EANM paediatric dosage card (55%), 4% the NACG, 4% specified by the local nuclear medicine society and 24% used a combination of these, while 13% used guidelines developed independently.

**Fig. 2:**
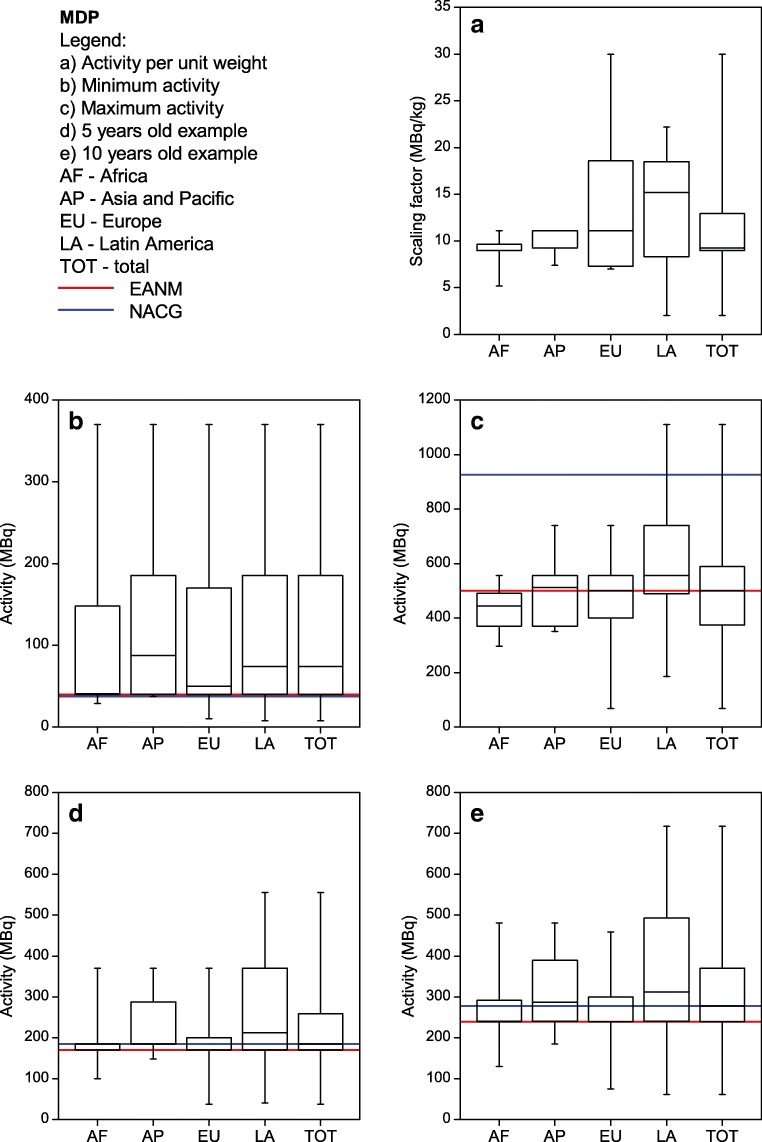
^99m^Tc MDP bone scans: median, box (25th and 75th percentiles) and whiskers (minimum and maximum values) for the scaling factor (**a**), minimum activity (**b**), maximum activity (**c**), activity for an hypothetical 5-year-old boy (20 kg, 110 cm tall) (**d**) and activity for an hypothetical 10-year-old girl (30 kg, 140 cm tall) (**e**). Values recommended by the EANM paediatric dosage card and the NACG are also reported as a reference

**Fig. 3:**
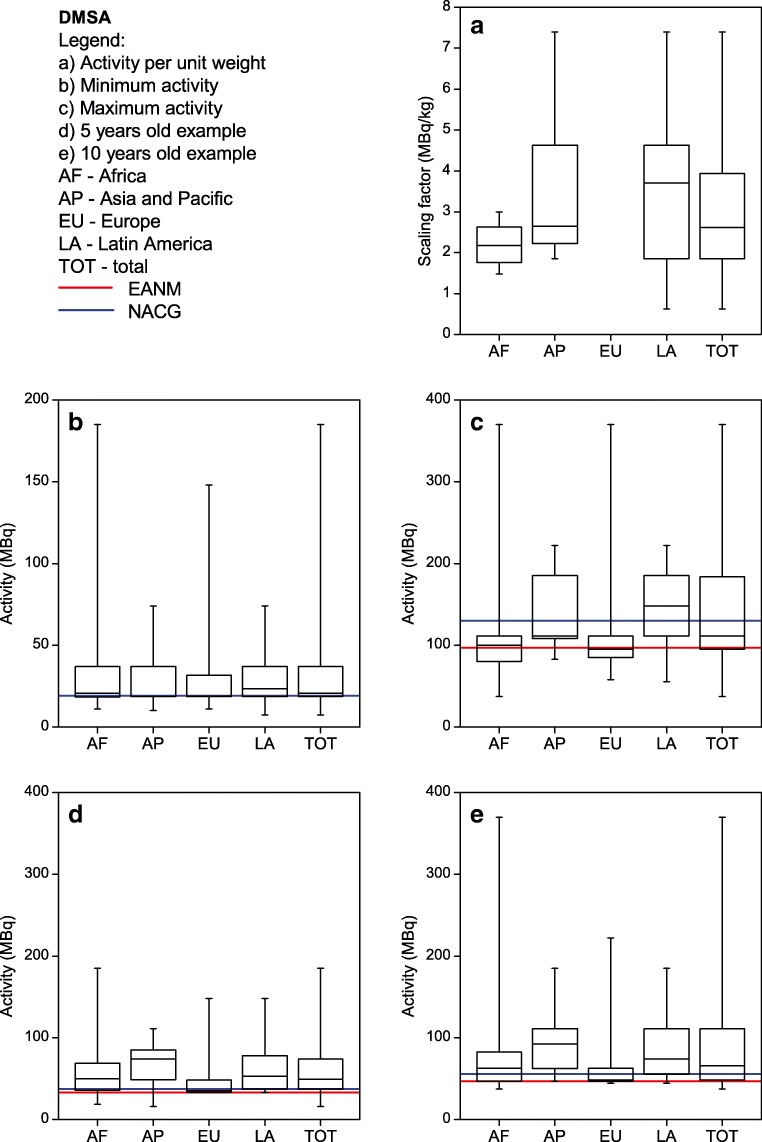
^99m^Tc DMSA renal cortical scans: median, box (25th and 75th percentiles) and whiskers (minimum and maximum values) for the scaling factor (**a**), minimum activity (**b**), maximum activity (**c**), activity for a hypothetical 5-year-old boy (20 kg, 110 cm tall) (**d**) and activity for an hypothetical 10-year-old girl (30 kg, 140 cm tall) (**e**). Values recommended by the EANM paediatric dosage card and the NACG are also reported as a reference

**Fig. 4:**
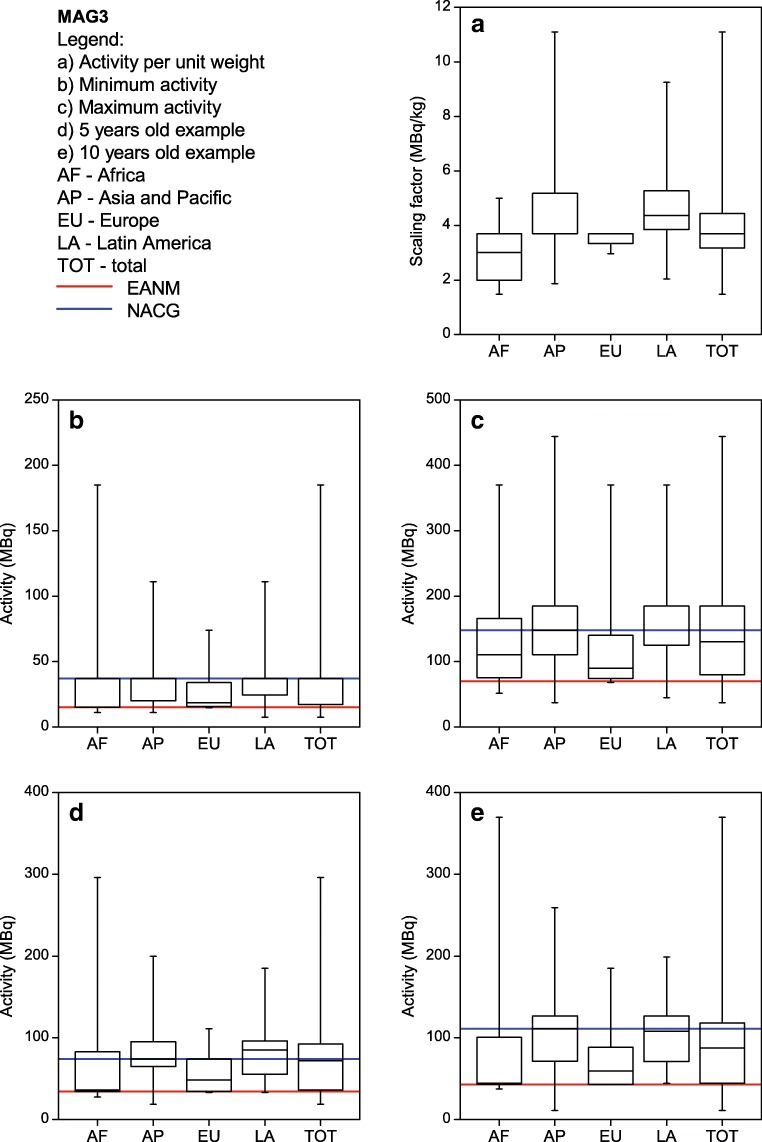
^99m^Tc MAG3 renograms: median, box (25th and 75th percentiles) and whiskers (minimum and maximum values) for the scaling factor (**a**), minimum activity (**b**), maximum activity (**c**), activity for an hypothetical 5-year-old boy (20 kg, 110 cm tall) (**d**) and activity for an hypothetical 10-year-old girl (30 kg, 140 cm tall) (**e**). Values recommended by the EANM paediatric dosage card and the NACG are also reported as a reference

**Fig. 5:**
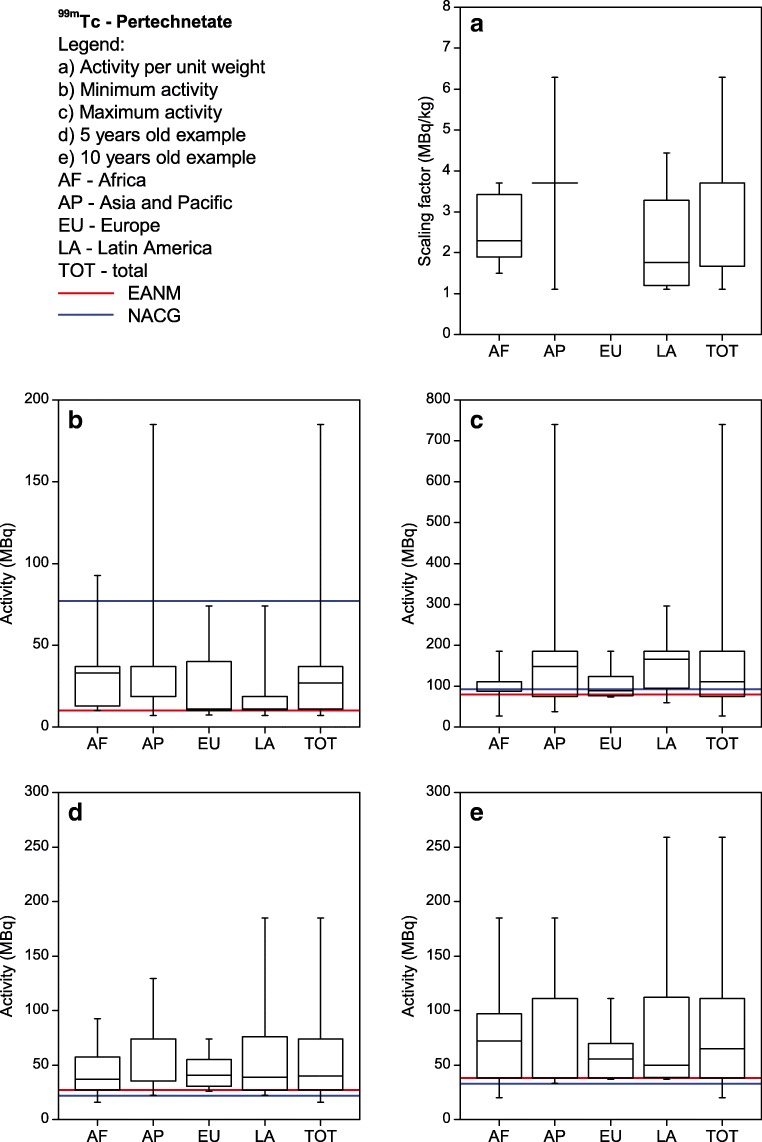
^99m^Tc pertechnetate thyroid scans: median, box (25th and 75th percentiles) and whiskers (minimum and maximum values) for the scaling factor (**a**), minimum activity (**b**), maximum activity (**c**), activity for an hypothetical 5-year-old boy (20 kg, 110 cm tall) (**d**) and activity for an hypothetical 10-year-old girl (30 kg, 140 cm tall) (**e**). Values recommended by the EANM paediatric dosage card and the NACG are also reported as a reference

**Fig. 6:**
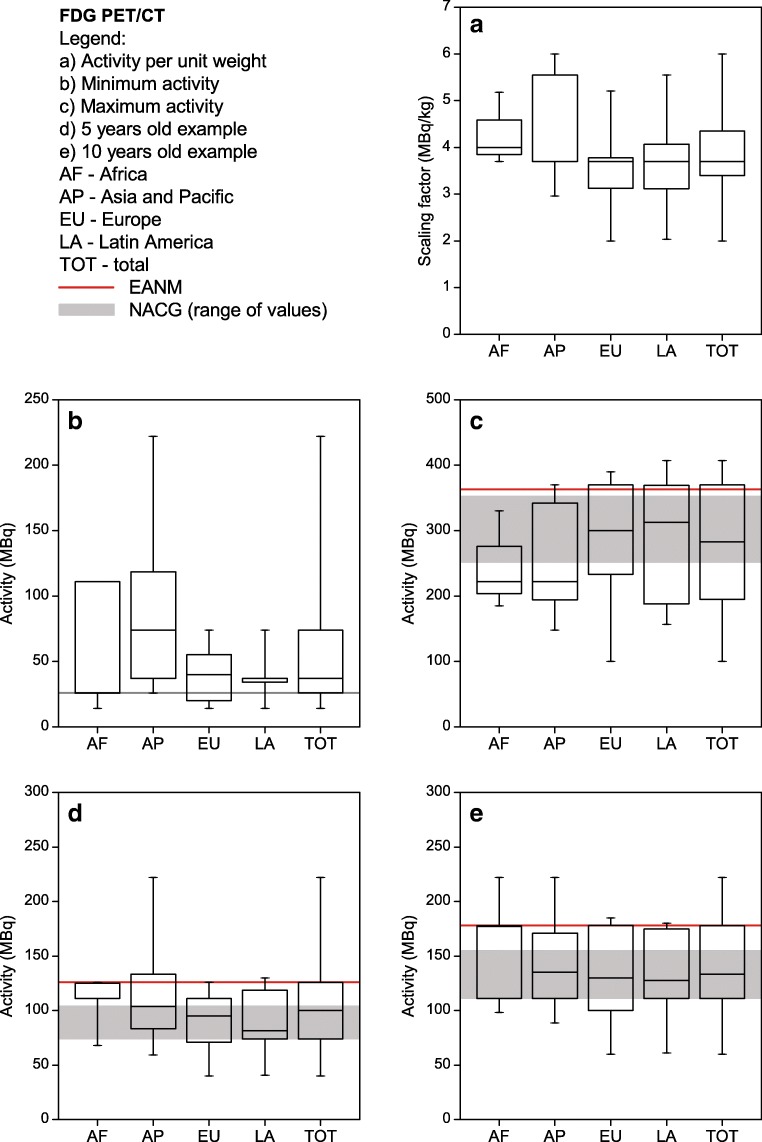
FDG PET/CT: median, box (25th and 75th percentiles) and whiskers (minimum and maximum values) for the scaling factor (**a**), minimum activity (**b**), maximum activity (**c**), activity for an hypothetical 5-year-old boy (20 kg, 110 cm tall) (**d**) and activity for an hypothetical 10-year-old girl (30 kg, 140 cm tall) (**e**). Values recommended by the EANM paediatric dosage card and the NACG are also reported as a reference

Each site listed their five most common single-photon nuclear medicine procedures performed in children. The most commonly listed procedures were ^99m^Tc-MDP bone scans, ^99m^Tc-DMSA renal cortical scans, ^99m^Tc-MAG3 renograms and ^99m^Tc pertechnetate thyroid scans. Therefore, the respective results for those four procedures as well as for FDG PET are shown in Figs. 2, 3, 4, 5 and 6. For each procedure, five box and whisker plots are provided: activity per body mass (a), minimum activity (b), maximum activity (c), activity for a 5-year-old boy (20 kg, 110 cm tall) (d) and activity for a 10-year-old girl (30 kg, 140 cm tall) (E). The median and 25th and 75th percentiles plus whiskers denoting the minimum and maximum values are shown as well as the values recommended by the EANM paediatric dosage card (noted as EANM) and the North American Consensus Guidelines (noted as NACG) where appropriate. The median and first and third quartiles for the five procedures including FDG PET are summarized in Table 2.

**Table 2:** Activity per body mass, minimum activity, maximum activity, activity for a 5-year-old boy (20 kg, 110 cm tall) and activity for a 10-year-old girl (30 kg, 140 cm tall) for FDG PET studies and the four most common single-photon procedures

	MDP	DMSA	MAG3	^99m^Tc pertechnetate	FDG
Scaling factor (MBq/kg)	[9.0; 9.3; 13.0]	[1.9; 2.6; 3.9]	[3.2; 3.7; 4.4]	[1.7; 3.7; 3.7]	[3.4; 3.7; 4.3]
Minimum activity (MBq)	[40; 74; 185]	[19; 21; 37]	[17; 37; 37]	[11; 27; 37]	[26; 37; 74]
Maximum activity (MBq)	[375; 500; 589]	[95; 111; 184]	[80; 130; 185]	[74; 111; 185]	[195; 283; 370]
5-year-old boy (EANM, NACG) (MBq)	[170; 185; 259] (170; 185)	[37; 49; 74] (33; 37)	[36; 72; 93] (34; 74)	[27; 40; 74] (27; 22)	[74; 100; 126] (126; 74-104)
10-year-old girl (EANM, NACG) (MBq)	[240; 278; 370] (240; 278)	[48; 66; 111] (47; 56)	[44; 87; 118] (43; 111)	[38; 65; 111] (38; 33)	[111; 133; 178] (178; 111-155)

Results are reported as [Q1; median; Q3]. For the two hypothetical paediatric patients, also the EANM and NACG recommended values are reported.

**Fig. 7:**
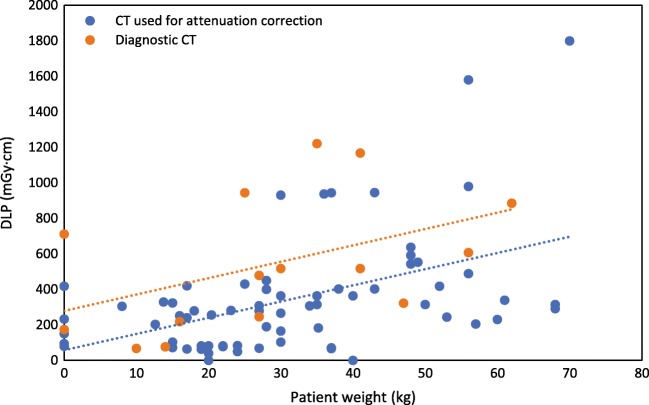
DLP values as a function of weight for the last five paediatric PET/CT scans performed by institutions using CT only for attenuation correction purposes (blue dots) or always acquiring a CT of diagnostic quality (orange dots). The two lines are a linear fit of the data to guide the eye

All 46 sites using PET/CT for paediatric patients corresponded to the questions regarding the use of CT in the context of PET/CT. More than 90% of these sites stated that they utilized tube current modulation in children. In addition, 17% stated that they always acquired CT of diagnostic quality as part of their paediatric PET/CT, 37% sometimes acquired diagnostic CT with PET/CT and 46% used the CT only for attenuation correction. About 11% said they always used CT contrast agents when performing PET/CT in children, 37% used contrast agents sometimes and 52% never used contrast agents. Figure 7 shows the resulting DLP values as a function of age. The results are reported only for those sites that indicated that they always acquired CT of diagnostic quality or only acquired CT for attenuation correction. One of the institutes using CT for diagnostic purposes reported DLP values up to 5300 mGy·cm, while another institute using CT only for attenuation correction purposes indicated DLP values as low as 3 mGy·cm. The two institutes confirmed these values and units; however, these data are considered as outliers and excluded from Fig. 7. The CT tube voltage varied from 80 to 130 kV_p_. Higher tube voltages were used indistinctly for all age/weight groups, while smaller tube voltages were used mainly for patients weighting less than 40 kg.

**Fig. 8:**
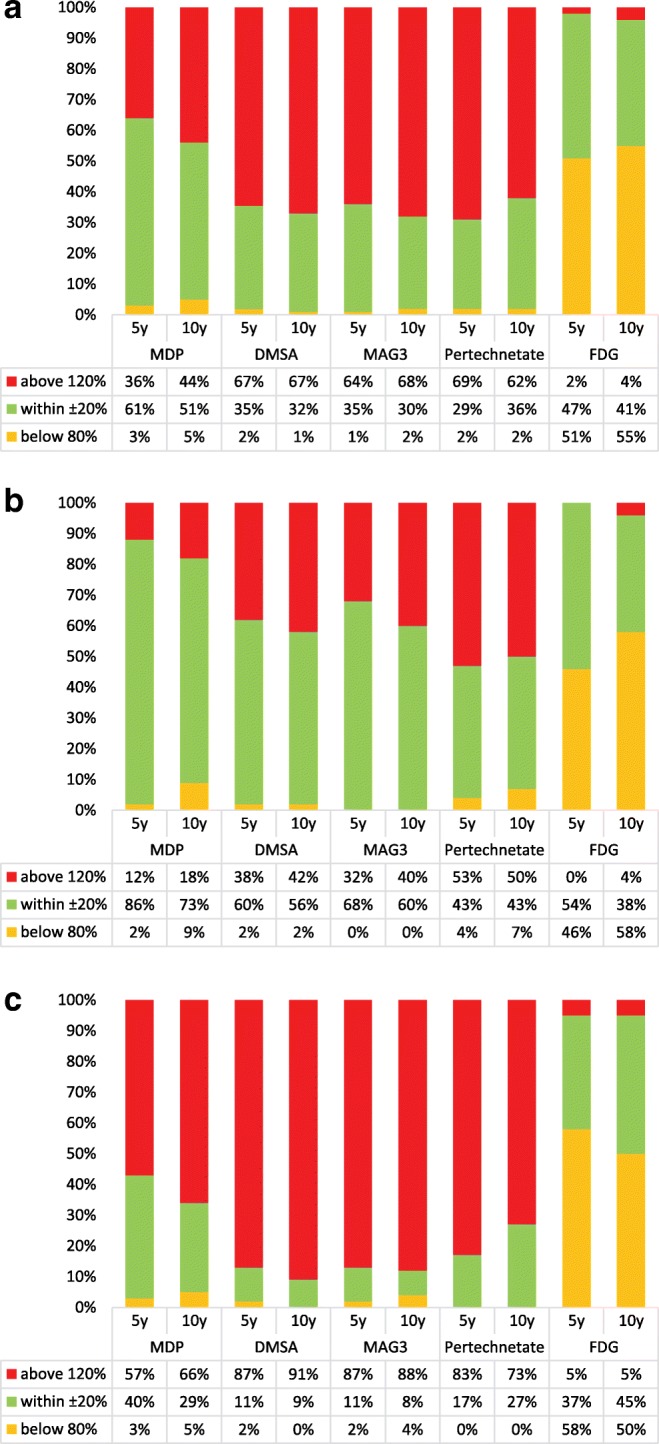
Percentage of centres that would administer an activity for the two examples which is below 80%, within ± 20% and above 120% of the EANM recommended activity: (**a**) all responders; (**b**) institutes following EANM recommendations and (**c**) institutes not following EANM recommendations

Figure 8 shows how the activities that the responders would administer to the two hypothetical paediatric patients relate to the recommendations of the EANM. The figure shows the results for (a) all responders, (b) institutes following EANM recommendations and (c) institutes not following EANM recommendations. By considering in basic compliance only administered activities which are within ± 20% of the EANM recommended activity, only 29 to 36% of the institutes would be adequate for the two examples for ^99m^Tc labelled DMSA, MAG3 and pertechnetate. However, the level of compliance for the same three examinations increases to 43–68% when only institutes following EANM recommendations are considered. The level of compliance for all institutes is higher for ^99m^Tc-MDP and FDG PET studies. It is also noted that for FDG PET studies, the percentage of institutes exceeding 120% of the EANM recommended activity is small (0–4%).

## Discussion

Overall, there was a high response rate to the survey. This is most likely attributable to the fact that all institutes surveyed had been contacted beforehand regarding the survey. As compared to the survey of the Nuclear Medicine Global Initiative project [9], the geographical spread of the responders was quite homogeneous for the different regions, with most of the countries having one participating institute and only three countries with more than five responders.

The majority of the sites stated that they used the EANM dosage card in its present version [3] or the NACG [4] or a combination of both. This, however, is not always reflected in the calculation of the administered activities for the hypothetical cases.

It can be seen from Figs. 2, 3, 4 and 5 that, for the four single-photon examinations considered, the first quartile for the two hypothetical examples is very close the EANM-recommended value, meaning that approximately 75% of the institutes would in fact administer an activity which is higher than the recommended one. The level of compliance to the EANM recommendations is somehow better for MDP studies, worsening more and more for DMSA, pertechnetate and MAG3. In the latter case, some institutes would administer activities which are up to nine times the EANM-recommended values.

Figure 8 shows how for ^99m^Tc labelled DMSA, MAG3 and pertechnetate, most of the sites (62–69%) would administer activities that are above 120% the EANM recommendations. The level of compliance however improves for those responders that declared to be following the EANM paediatric dosage card.

A comparison of the results of this survey with respect to the two hypothetical cases shows, for MDP, a similar spread of calculated administered activities as the NMGI survey [9]. The largest spread of data was observed for LA, whereas for other regions like AF and EU, the interquartile range was lower and the respective medians were in line with the values of the EANM dosage card. For DMSA, most centres would apply higher activities as compared to the respective dosage recommendations. This was also observed in the NMGI study for this radiopharmaceutical. A possible explanation for this observation is that the sites aim at shorter scanning times in order to reduce motion artefacts.

For MAG3 the differences between the respective recommendation are more pronounced than for the other pharmaceuticals considered in this survey. Most of the proposed activities are within the values of the NACG and the EANM recommendation. For MAG3, the spread of the data is reduced compared to the results of the NMGI survey. For ^99m^Tc pertechnetate, the NMGI published no data; therefore a comparison is not possible.

For FDG PET, the spread of the data according to this survey is much lower as compared to the NMGI data. In addition, no activities higher than 230 MBq were reported, which is not the case for the NMGI data, where activities of up to 400 MBq were conveyed by several sites. An explanation could be that the way of using FDG PET is better harmonized worldwide as compared to single-photon scanning.

For FDG PET, the activity values reported by the participants showed, in general, good compliance with the international recommendations. However, the data on the use of the CT (not reported by the NMGI) showed highly heterogeneous results concerning the kV_p_ applied as well as the DLPs. The kV_p_ ranged from 80 to 130 kV_p_, although there are some recommendations for paediatric CTs that recommend reducing the kV_p_ for paediatric exams (e.g. see the paediatric protocols published by AAPM [12]). While some institutions are reducing the tube voltage for smaller children (below 40 kg), most of the centres used highest voltages (120 or 130 kV_p_) for all the patients regardless of patient age or weight. This observation did not depend on whether the study was performed for attenuation correction only or as a diagnostic scan with or without a contrast agent. In general, as was expected, however with some exceptions, the DLP levels were higher for diagnostic scans than for attenuation correction scans. Interesting to note is that DLP values of 750 mGy·cm have been proposed for whole body PET/CT in France for adults [13]. Data from a survey in Australia and New Zealand showed mean DLPs for adults between 474 mGy·cm in Australia and 1319.05 mGy·cm in New Zealand [14].

## Conclusions and recommendations

Overall, the survey shows, that in most parts of the world, efforts have been undertaken to comply either with the EANM dosage card or the NACG.

The following recommendations for future actions are derived from the observations of this study:

- The reasons for the general use of higher activity values for ^99m^Tc labelled DMSA, MAG3 and pertechnetate as compared to the international recommendations need further attention. In the case of DMSA studies, data could be acquired in dynamic mode and reframed to a single image after discarding frames which are blurred due to patient motion. This approach suggested in the EANM guideline [15] allows for a longer total study duration with reduced administered activity.
- During the data analysis of the survey, we observed in several cases an incorrect usage of the EANM paediatric dosage card when the calculation is performed step by step. For this reason, the authors suggest the use of the on-line tool [16] or the available smartphone app which are less prone to errors than manual calculations.
- For those institutes that are following international recommendations, the adherence to administered activities that are considered adequate was generally larger. International recommendations are therefore a vital tool towards standardization in paediatric imaging.
- The training of medical physics experts needs to be extended to include CT protocols in FDG PET studies in order to reduce unnecessary patient exposure. An example is the use of a diagnostic scan for attenuation correction purposes only. It was also acknowledged from the data analysis and communications with the responders that health professionals involved in the survey were not always sufficiently acquainted with dose metrics used in CT such as CTDI_vol_ and DLP.
- As in some parts of the world, the spread of the data was more pronounced and more efforts need to be undertaken to promote the use of international standards for determining the activities to be administered in children.
